# Combination of Cytokine Responses Indicative of Latent TB and Active TB in Malawian Adults

**DOI:** 10.1371/journal.pone.0079742

**Published:** 2013-11-18

**Authors:** Yun-Gyoung Hur, Patricia Gorak-Stolinska, Anne Ben-Smith, Maeve K. Lalor, Steven Chaguluka, Russell Dacombe, T. Mark Doherty, Tom H. Ottenhoff, Hazel M. Dockrell, Amelia C. Crampin

**Affiliations:** 1 Department of Immunology and Infection, Faculty of Infectious and Tropical Diseases, London School of Hygiene & Tropical Medicine, London, United Kingdom; 2 Department of Infectious Disease Epidemiology, Faculty of Epidemiology & Population Health, London School of Hygiene & Tropical Medicine, London, United Kingdom; 3 Karonga Prevention Study, Chilumba, Malawi; 4 GSK, Nykær 68 Copenhagen, Denmark; 5 Department of Infectious Diseases, Leiden University Medical Center, Leiden, The Netherlands; University of Missouri-Kansas City, United States of America

## Abstract

**Background:**

An IFN-γ response to *M. tuberculosis*-specific antigens is an effective biomarker for *M. tuberculosis* infection but it cannot discriminate between latent TB infection and active TB disease. Combining a number of cytokine/chemokine responses to *M. tuberculosis* antigens may enable differentiation of latent TB from active disease.

**Methods:**

Asymptomatic recently-exposed individuals (spouses of TB patients) were recruited and tuberculin skin tested, bled and followed-up for two years. Culture supernatants, from a six-day culture of diluted whole blood samples stimulated with *M. tuberculosis*-derived PPD or ESAT-6, were measured for IFN-γ, IL-10, IL-13, IL-17, TNF-α and CXCL10 using cytokine ELISAs. In addition, 15 patients with sputum smear-positive pulmonary TB were recruited and tested.

**Results:**

Spouses with positive IFN-γ responses to *M. tuberculosis* ESAT-6 (>62.5 pg/mL) and TB patients showed high production of IL-17, CXCL10 and TNF-α. Higher production of IL-10 and IL-17 in response to ESAT-6 was observed in the spouses compared with TB patients while the ratios of IFN-γ/IL-10 and IFN-γ/IL-17 in response to *M. tuberculosis*-derived PPD were significantly higher in TB patients compared with the spouses. Tuberculin skin test results did not correlate with cytokine responses.

**Conclusions:**

CXCL10 and TNF-α may be used as adjunct markers alongside an IFN-γ release assay to diagnose *M. tuberculosis* infection, and IL-17 and IL-10 production may differentiate individuals with LTBI from active TB.

## Introduction

Tuberculosis (TB) remains a global health problem with approximately one third of the world population thought to be infected with *M. tuberculosis* (*M. tb*) [1]. Among infected individuals, only about 10% progress to active disease and the remaining 90% maintain a latent TB infection (LTBI) without clinical TB symptoms [2]. These individuals with LTBI can remain healthy for decades or, in an unknown proportion, resolve infection spontaneously but have a significantly elevated risk of developing TB during their life time. Therefore, a major challenge of TB control includes diagnosis and treatment of latently-infected individuals who may act as a reservoir for new TB cases, particularly in areas where TB is endemic.

The IGRA (IFN-γ release assay) using the immune response to the *M. tb* virulence factors, early secreted antigen target, 6 kDa (ESAT-6) and culture filtrate protein, 10 kDa (CFP-10) to diagnose *M. tb* infection has been developed to overcome the drawback of the tuberculin skin test which includes false positive reactions due to cross-reactivity between tuberculin and antigens from environmental mycobacteria and BCG [3]. However, despite the specificity of these antigens, IGRA does not distinguish between latent TB infection and active TB disease [4]. Thus, there is an urgent need to identify better immunological biomarkers of TB infection and disease status.

Transmission of TB from household contacts has been identified as an important source of infection in Malawi [5]. An epidemiological study by the Karonga Prevention Study (KPS) in rural Malawi showing that 5% of adult TB cases reported their spouses with previous smear positive TB [6] indicates that spouses of TB patients are at particularly high risk of *M. tb* infection and disease, presumably by virtue of intensive intimate exposure to TB patients through daily contact and nursing. The prevalence of tuberculin skin test (TST) “positivity” was higher in the recruited spouses of TB patients than in community controls using two cut-offs (≥10 and ≥15 mm of indurations) [7], as was the median size of TST induration. The high rate of TST positivity and conversion in spouses compared with community controls confirms that the spouses of TB patients are a high risk group for *M. tb* infection.

The current study was therefore designed to look at cytokine responses to a panel of mycobacterial antigens including *M. tb* ESAT-6 and *M. tb*-derived purified protein derivative (PPD) in the same cohort of 148 spouses of TB patients, using a previously-described whole blood assay (WBA) [8]. We hypothesized that the individuals with presumed recent *M. tb* infection who remained healthy, must have a state (of uncertain duration) analogous to protective immunity against the pathogen as they have not rapidly progressed to disease and would therefore have different cytokine and chemokine responses to *M. tb* antigens compared to TB patients. We performed IFN-γ ELISAs in the spouses without TB and HIV infection, and in addition, determined the production of the cytokines; IL-10, IL-13, IL-17, CXCL10/IP-10 and TNF-α in response to PPD and ESAT-6 antigens. These responses were compared to those in 15 patients with sputum smear-positive pulmonary TB.

## Materials and Methods

### Prospective recruitment of spouses of index TB patients

The spouses selected as TB contacts for this study were a sub group derived from a large cohort recruited for an epidemiological study for which sputum smears, cultures and immunological assays were conducted at KPS [7]. From November 2002 to July 2005, smear positive TB patients were asked about co-residence with any spouse since onset of cough and their spouses were recruited six weeks after the index case started treatment by direct contact on the TB ward or by visiting the household. Eligibility of spouses was verified and outcome details of the index case were collected. Informed written consent was obtained for interview and all tests; TST (Mantoux method with 2 IU intradermal RT23), HIV counselling and testing, syphilis and pregnancy testing, CD4 count, clinical examination and blood sampling for immunological testing. Spouses were invited to attend the hospital for clinical review and chest X-ray to exclude active TB. Those presenting with persistent cough provided a sputum sample for testing [7]. Amongst the 148 recruited spouses, 143 spouses had no active TB at baseline and 89 were HIV negative. The spouses were followed up at 6, 12 and 24 months after the baseline date with TST and blood collection to examine the IFN-γ immune responses to *M. tb* antigens such as PPD and ESAT-6. None of the spouses without TB at baseline developed TB during the two years of active follow-up [7]. However, 7 spouses are known to have progressed to active TB in the subsequent period [7].

Ethical permission for this study to look at the risk of infection and disease progression in spouses of TB patients was granted by the National Health Science Research Committee (NHSRC) in Malawi (#01138) and the ethics committee of the London School of Hygiene & Tropical Medicine (LSHTM) (#745a) [6].

### Diluted WBA and IFN-γ ELISA

A WBA and quantitative IFN-γ ELISA were performed as previously described [8]. Briefly, heparinized blood was diluted by 1 in 5, with RPMI 1640 supplemented with penicillin and streptomycin plus 2 mM L-glutamine (Gibco BRL, Paiseley, UK). A total of 100 µL was added into each well with 100 µL of each PPD (RT49; Lot204; Statens Serum Institute (SSI), Copenhagen, Denmark] at a final concentration of 5 µg/mL, ESAT-6 (SSI) at a final concentration of 10 µg/mL, or PHA (149781KA; Difco Laboratories, Oxford, UK) at a final concentration of 5 µg/mL and RPMI 1640 supplemented with 2 mM L-glutamine (Gibco BRL). After a 6-day incubation at 37°C, the culture supernatant was harvested and the production of IFN-γ was measured in culture supernatant using commercial antibody pairs (Pharmingen, San Diego, California) [8]. Recombinant IFN-γ (Pharmingen) was used for the standard curve with a range of 31 – 2000 pg/mL and a “positive” IFN-γ response was defined as >62.5 pg/mL [8].

### Collection of spouse samples for other cytokine ELISAs

The culture supernatant samples obtained from the prospectively recruited spouses [7] have been stored at −80°C for 4 years since the WBA and IFN-γ ELISA were carried out. Samples from spouses with HIV were excluded from this study and 150 archived samples at all of the follow-up stages as well as the baseline were collected for further research. These 150 archived culture supernatant samples are composed of the samples obtained from 40 subjects at baseline, 49 at 6 months, 39 at 12 months and 22 at 24 months follow-up. Some of the samples were unavailable at baseline or follow-up as those were previously used up for other assays. Due to the limitation of the samples which were available from the same subjects, we performed our study focusing on the groups at baseline and each follow-up rather than on individual follow-up. The subjects consists of 33% of males and 67% of females aged 40 on average. The archived samples include one control cultured with RPMI medium only and 3 stimulated supernatants i.e. PPD, ESAT-6 and PHA from each individual resulting in a total of 600 culture supernatant samples that were available to test in this study.

### Recruitment of Malawian TB patients and IFN-γ ELISA

TB patients aged between 18 and 50 years were recruited (mean age: 41, 40% males and 60% females), at diagnosis or within the first three months of treatment, and used as TB disease controls. HIV positive individuals, anyone on steroids or chemotherapy and those who had any form of cancer or diabetes were excluded. Any special population i.e. pregnant women, prisoners, and mentally and physically disabled subjects were also excluded from this study. Information sheets, including those translated into local languages, were prepared for TB patients at Karonga District Hospital and Chilumba Rural Hospital in Malawi. The study was explained to the patients and written consents were obtained from all of the study participants. Study samples and questionnaires were identified by a unique blood number and study number for confidentiality in a specimen form. Ethical permission for 15 TB patients was granted by the LSHTM ethics committee (#5929) and NHSRC in Malawi (#866) after the review of ethics application forms including research proposal, consent forms and information sheets.

The diluted WBA for TB patients was performed as previously described [8] using PPD (RT50; Lot219; SSI) at a final concentration of 5 µg/mL, ESAT-6/CFP-10 [9–10, Bill and Melinda Gates Foundation Grand Challenge 6 (BMGF GC6) project; batch 040101] at a final concentration of 10 µg/mL, PHA (Lot017k4029; Sigma-Aldrich) at a final concentration of 5 µg/mL and RPMI 1640 supplemented with 1% L-glutamine (Sigma-Aldrich). After a 6-day incubation at 37°C, the culture supernatant was harvested and the production of IFN-γ was measured in culture supernatant. The IFN-γ ELISA protocol was slightly modified using the standard sigmoid curve for TB patients [11].

### Cytokine ELISAs in the samples of spouses and TB patients

A total of 150 samples were selected for 5 different cytokine ELISAs and each sample included cultures stimulated with, PPD or ESAT-6 and PHA and RPMI-only controls which were used for whole blood assays with 6-day incubation. Thus, 4 culture supernatant samples obtained from each of 150 spouses (n = 600) were used for these ELISAs, and 56 samples were used for a comparison test to confirm the results between the plates using different volumes of samples, standards and positive controls. To control for interplate and intraplate variation, the positive controls for each cytokine ELISA were prepared with culture supernatant stimulated with PHA for IL-10, IL-13, IL-17, CXCL10, and LPS for TNF-α.

These samples had limited volumes for the further assays, and a multiple transfer technique of the samples was applied for the 5 cytokine ELISAs. Samples were loaded on the IL-13 plate first and transferred to IL-10, CXCL10, IL-17 and TNF-α plates in turn according to the expected limit of detection for each assay (from lowest to highest). The volume of samples transferred started at 100 µL for IL-13, 95 µL for IL-10, 90 µL for CXCL10, 85 µL for IL-17 and 80 µL for TNF-α. The volume of standard and positive control followed the same volumes of samples for each different cytokine ELISA. The ELISAs for IL-10, IL-13, IL-17, TNF-α and CXCL-10 were performed using the kits purchased from R & D Systems (DuoSet ELISA Development System). The positive responses were defined as values which are twice the limit of detection of the assays in order to minimize false positive responses; >31 pg/mL for IL-17 and TNF-α, >62.5 pg/mL for IL-10 and CXCL10, and >188 pg/mL for IL-13.

### Statistical analysis

Spearman's rank correlation coefficient was calculated to measure the association of cytokine responses between intact (untransferred) and transferred samples in 5 different cytokine ELISAs and the correlation between TST induration size and cytokine responses. The difference of median responses of cytokine production among three groups depending on the IFN-γ responses to ESAT-6 in spouses and TB patients were analysed by one way ANOVA and Dunn's multiple comparison tests.

## Results

### Comparison of cytokine responses between intact and transferred samples

Due to limited sample volumes for the cytokine ELISAs, culture supernatant samples were transferred sequentially from one cytokine ELISA plate to another cytokine ELISA plate. IL-13 was the first cytokine tested in the sequence, and simply received untreated supernatant. However, although ELISA capture should be specific for the cytokine to be tested, there is the possibility that the ELISA results could be affected by non-specific protein loss (for example by adsorption). Therefore, all of the subsequent ELISAs where supernatant was transferred (IL-10, IL-17, TNF-α and CXCL10) were compared using 56 culture supernatant samples to test the influence of transferring samples on cytokine responses. Spearman's rank correlation coefficients were above 0.9 (P<0.001) in all of the cytokine responses, indicating that there is a very strong association of the cytokine responses when comparing transferred samples or sample used directly on one plate. This indicates that the multiple transfer of samples from one assay plate to the next did not affect the interpretation of the results (Figure S1).

### Distribution of cytokine responders at baseline and follow-up in spouses

**Figure 1:**
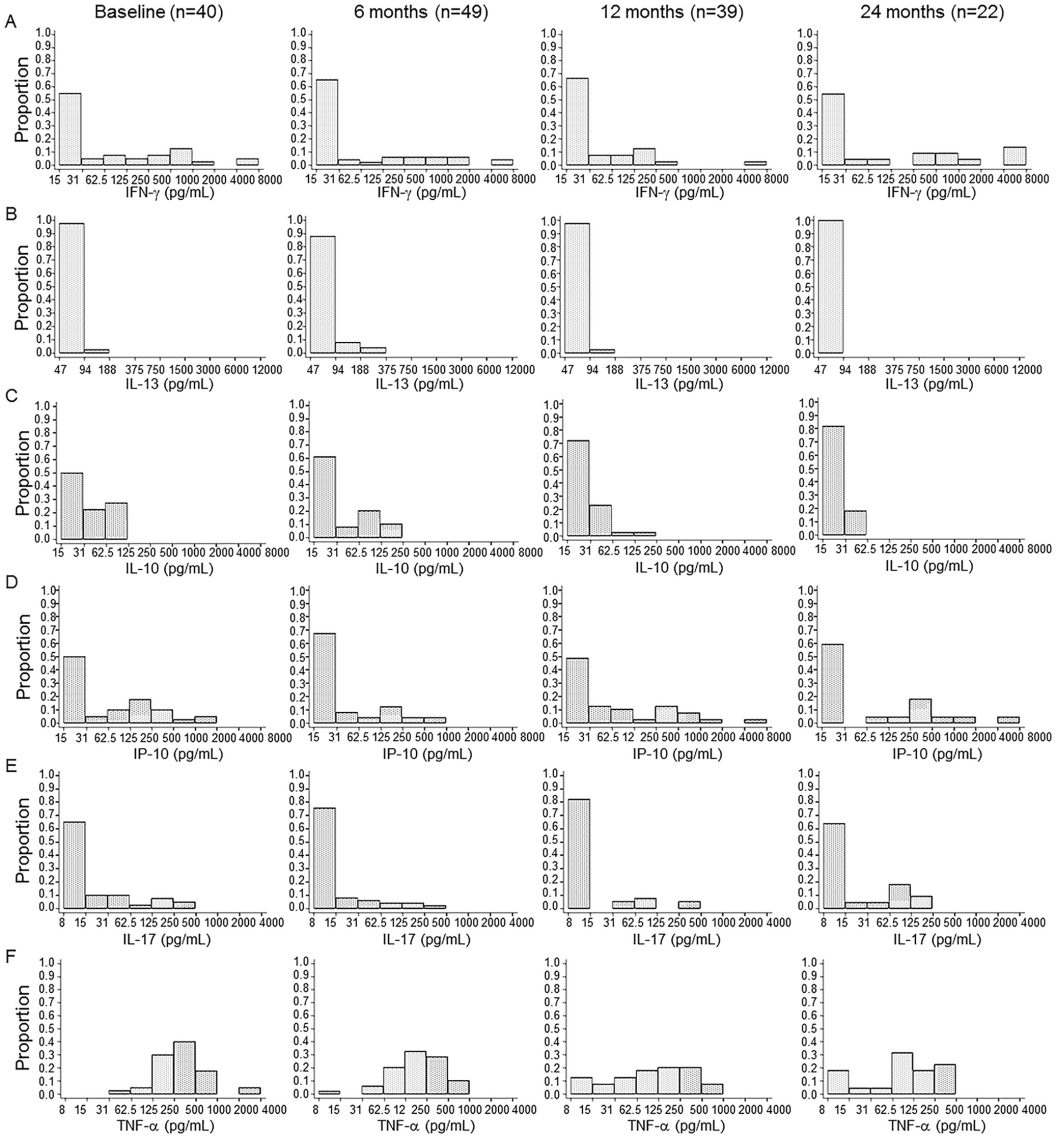
The proportion of cytokine responders to ESAT-6 at baseline and follow-up time points. The proportion of responders producing IFN-γ (A), IL-13 (B), IL-10 (C), CXCL10 (D), IL-17 (E) and TNF-α (F) in response to ESAT-6 was analysed in 40 spouses at baseline, 49 at 6 months, 39 at 12 months and 22 at 24 months follow-up. There were no significant differences in the proportions of IFN-γ responders between baseline and 24 months follow-up. Most of spouses did not produce IL-13 in responses to ESAT-6 at baseline, and this did not change over the 24 months follow-up. The proportions of IL-10 and TNF-α responders to ESAT-6 gradually declined at 6, 12 and 24 months.

**Figure 2:**
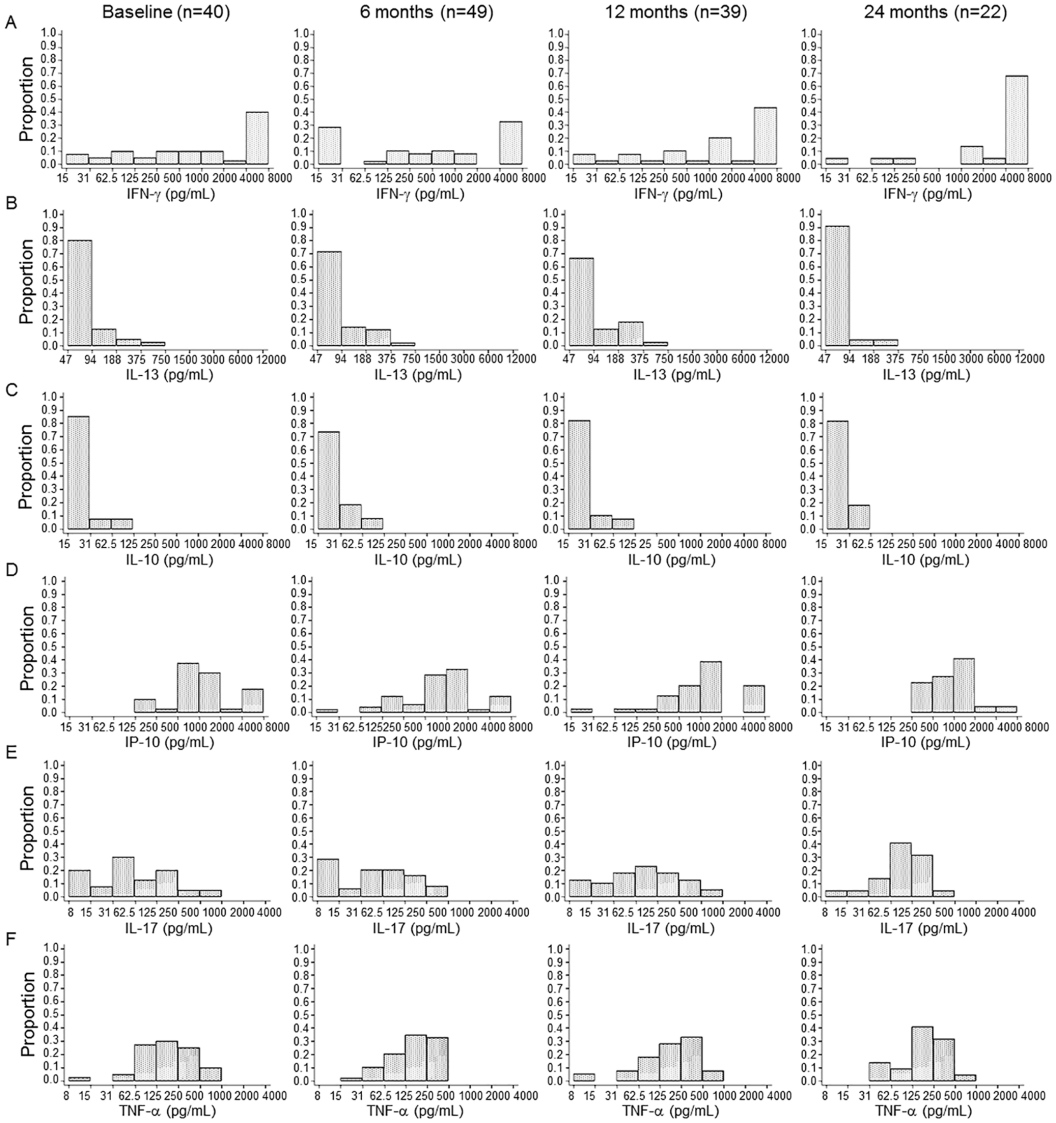
The proportion of cytokine responders to PPD at baseline and follow-up time points. The proportion of responders producing IFN-γ (A), IL-13 (B), IL-10 (C), CXCL10 (D), IL-17 (E) and TNF-α (F) in response to PPD was analysed in 40 spouses at baseline, 49 at 6 months, 39 at 12 months and 22 at 24 months follow-up. The majority of the spouses did not produce IL-13 and IL-10 in response to PPD at baseline or 24 months later while high levels of IFN-γ, CXCL10, IL-17 and TNF-α were detected. There were no significant changes in the proportions of cytokine responders throughout the follow-up period.

The proportion of individuals producing IFN-γ, IL-13, IL-10, CXCL10, IL-17 and TNF-α upon ESAT-6 (Figure 1) and PPD (Figure 2) stimulation was analysed at baseline and follow-up. There were no significant changes in proportions of IFN-γ responders to ESAT-6 and PPD in the spouses who were prospectively recruited at baseline and follow-up time points [12]. In this study, the distribution of IFN-γ responders was reanalysed in HIV-negative subjects who were involved in the analysis of additional cytokines: 40 subjects at baseline, 49 at 6 months, 39 at 12 months and 22 at 24 months follow-up. In response to ESAT-6, 16 of 40 spouses showed positive IFN-γ responses (>62.5 pg/mL) at baseline (Figure 1A) while 35 of the 40 spouses showed positive IFN-γ responses to PPD (Figure 2A). Although the proportion of the responders showing a higher level of IFN-γ production (>500 pg/mL) to PPD was increased at 24 months (19/22) compared to baseline (25/40), there were no significant differences of the proportions of responders at 24 months in response to PPD and ESAT-6 (P>0.05, Figure 2A and 1A). The magnitude of IFN-γ responses to ESAT-6 was not significantly changed from the baseline to each follow-up time point while the median value of IFN-γ in response to PPD gradually increased (from 517 to 4000 pg/mL) after 6 months follow- up (data not shown).

All of the spouses showed positive TNF-α responses (>31 pg/mL) to ESAT-6 at baseline and the proportion of responders who produced high levels of TNF-α (>250 pg/mL) declined according to the follow-up time (Figure 1F). In contrast, most of the spouses did not produce IL-13 at baseline and follow-up time points (<188 pg/mL) (Figure 1B). The percentage of IL-10 responders (>62.5 pg/mL) was 27.5% (11/40) at baseline but the proportion gradually decreased to 5.1% (2/39) (P<0.05) and 0% (P<0.01) at 12 and 24 months follow-up respectively (Figure 1C). The proportion of CXCL10 responders to ESAT-6 (>62.5 pg/mL) was 40% at baseline but there was no significant difference of the proportion throughout the follow-up stages (P>0.05, Figure 1D). The percentage of IL-17 responders (>31 pg/mL) was 27.5% (11/40) at baseline and the proportion was slightly decreased to 18.4% (9/49) and 17.9% (7/39) at each 6 and 12 month time point (P>0.05, Figure 1E). In response to PPD, the majority of the spouses tested showed CXCL10 (40/40), IL-17 (29/40) and TNF-α responses but only 3 of 40 spouses showed IL-13 and IL-10 responses at baseline (Figure 2). There were no significant differences in the patterns of CXCL10 and TNF-α production throughout the follow-up period (P>0.05, Figure 2).

### Immune responses based on IFN-γ responses to ESAT-6

**Figure 3:**
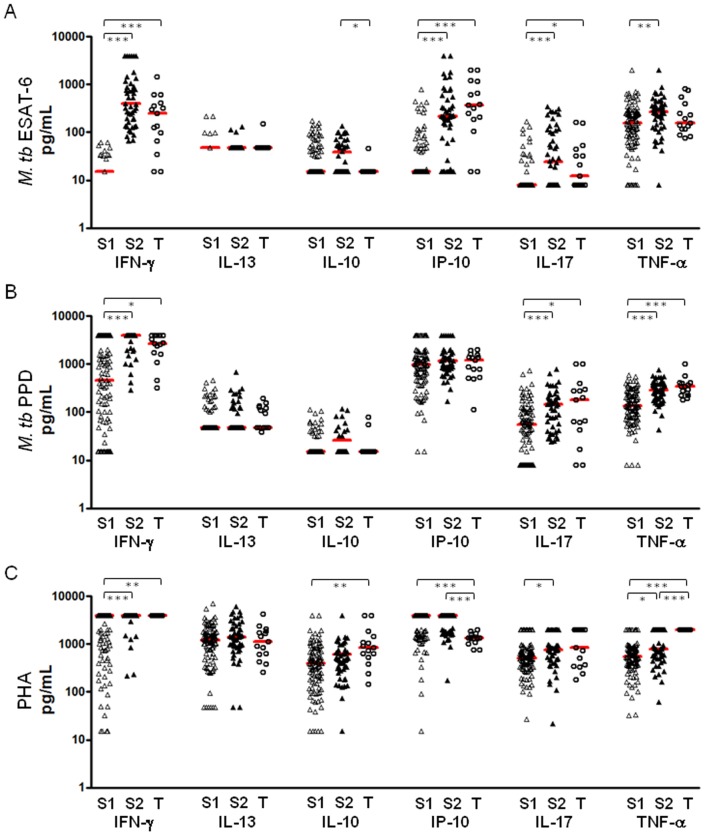
Cytokine responses in spouses and TB patients. The cytokine responses to ESAT-6 (A), PPD (B) and PHA (C) were analysed in the spouses who showed negative IFN-γ responses to ESAT-6 (<62.5 pg/mL, n = 102; indicated as S1), the spouses with positive IFN-γ responses (>62.5 pg/mL, n = 48; indicated as S2) and TB patients (n = 15; indicated as T). The S2 contacts and TB patients showed significantly higher production of CXCL10, IL-17 and TNF-α production in response to ESAT-6 compared with the S1 contacts. In response to PPD, IL-17 and TNF-α production was also significantly higher in the 2 groups compared with the group of S1. However, only IL-10 and IL-17 differentiated the S2 spouses from TB patients by showing higher production in the S2 group than TB patients. In response to PHA, all cytokines were highly detected in 3 groups. Median responses are marked with horizontal bars. (* P<0.05, ** P<0.01, *** P<0.001)

The spouses were divided into two groups based on the IFN-γ responses to ESAT-6 and 48 spouses showing positive IFN-γ responses (>62.5 pg/mL and designated “S2”) were considered to be those who were most likely to have LTBI compared with 102 spouses with negative IFN-γ responses (<62.5 pg/mL, designated “S1”). The cytokine responses including IL-13, IL-10, CXCL10, IL-17 and TNF-α in response to ESAT-6, PPD or PHA were compared among the S1 spouses, the S2 spouses and TB patients. As would be predicted, median production of IFN-γ and CXCL10 in response to ESAT-6 was significantly higher in the S2 spouses compared with the S1 group (P<0.001). However, these cytokine responses did not differentiate between the S2 contacts and patients (Figure 3A) who both produced significantly more IFN-γ and CXCL10 than the S1 spousal contacts (P<0.001). However, production of IL-10 was significantly higher only in the S2 spouses compared with both TB patients and S1 contacts (P<0.05, Figure 3A). IL-17 production was also higher in response to ESAT-6 in the S2 spouses compared to the other groups with or without significance (P<0.001; S1, P>0.05; TB, Figure 3A). IL-13 production in response to ESAT-6 was not significantly different between any of the groups, and although the spouses had higher levels of IL-10 than the TB patients, this was only significant for the S2 spouse group (Figure 3A). In response to PPD, IFN-γ, IL-17 and TNF-α production were all significantly higher in both S2 spouses and TB patients compared with those with negative IFN-γ responses to ESAT-6 while none of the cytokines tested differentiated between the S2 and TB groups (Figure 3B). All cytokines were highly produced in response to PHA, suggesting no effect of non-specific immunosuppression on cytokine responses to ESAT-6 and PPD (Figure 3C).

### Analysis of immune responses by the ratio of cytokine responses

**Figure 4:**
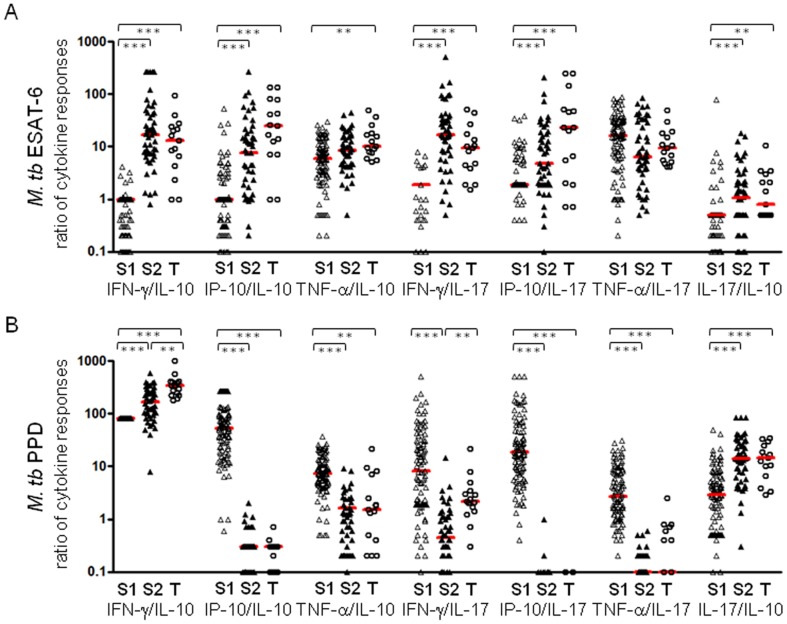
The ratio of cytokine responses in spouses and TB patients. The ratio of cytokine responses to ESAT-6 (A) and PPD (B) were analysed in the spouses who showed negative IFN-γ responses to ESAT-6 (<62.5 pg/mL, n = 102; indicated as S1), the spouses with positive IFN-γ responses (>62.5 pg/mL, n = 48; indicated as S2) and TB patients (n = 15; indicated as T). The ratio of IFN-γ/IL-10, CXCL10/IL-10, TNF-α/IL-17, IFN-γ/IL-17 and IL-17/IL-10 differentiated the S2 and TB groups from S1 in responses to both ESAT-6 and PPD. None of the ratios differentiated between the S2 and T groups upon ESAT-6 stimulation while IFN-γ/IL-10 and IFN-γ/IL-17 in response to PPD distinguished S2 from TB patients. Median ratio of cytokine responses is marked with horizontal bars. (* P<0.05, ** P<0.01, *** P<0.001)

Since cytokines can modulate each other's effects and are rarely produced in isolation, the cytokine responses to ESAT-6 and PPD were further analysed by the ratio of cytokine responses between the groups of spouses and TB patients. This is particularly important when looking at the production of pro- and anti-inflammatory cytokines. The ratio of IFN-γ/IL-10, CXCL10/IL-10, IFN-γ/IL-17, CXCL10/IL-17 and IL-17/IL-10 in response to ESAT-6 was significantly higher in both S2 spouses and TB patients compared with the S1 contact group (Figure 4A). The ratio of TNF-α/IL-10 was also significantly higher in TB patients than in the S1 group (P<0.01) while none of the ratios in cytokine responses to ESAT-6 differentiated between S2 contacts and TB patients (Figure 4A). On the other hand, the ratio of IFN-γ/IL-10 and IFN-γ/IL-17 in responses to PPD discriminated the TB patient group from the S2 spouses with significantly higher responses in TB patients (P<0.01) (Figure 4B). The median ratio of IFN-γ/IL-10 response in S2 spouses and TB patients was 165 and 338 respectively, and 0.45 and 2.2 for IFN-γ/IL-17 in each group.

### TST induration in spouses and association between TST size and immune responses

**Figure 5:**
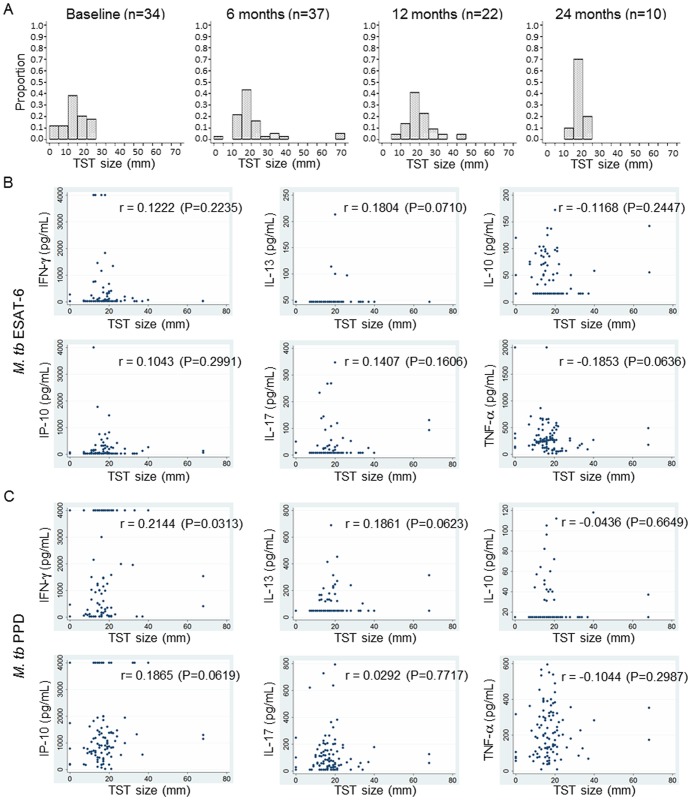
TST indurations and association between TST size and cytokine responses. A. TST sizes were obtained from 34 spouses at baseline, 37 spouses at 6 months, 22 spouses at 12 months and 10 spouses at 24 months follow-up time point. More than 75% of spouses already had above 10 mm of TST induration at baseline. The proportion of those who had more than 10 or 15 mm of TST induration was increased according to the follow-up time while the proportion of those with below 10 mm of TST induration was decreased. None of the subjects showed a TST induration below 10 mm at the 24 months follow-up but about 80% of these had more than 15 mm of TST induration. B–C. None of the cytokine production in response to ESAT-6 (B) and PPD (C) was correlated with TST size in 101 spouses by Spearman's rank correlation test. Spearman's rank correlation coefficients (r) and P values are indicated in each graph.

Those individuals who have TST induration of more than 10 and 15 mm are generally assumed to be infected with *M. tb*. The information on TST induration size was obtained for 34 spouses at baseline, 37 spouses at 6 months, 22 spouses at 12 months and 10 spouses at 24 months follow-up time point. The majority of spouses already had TST induration of more than 10 mm at baseline (Figure 5A). The proportion of spouses with a TST response greater than 10 mm increased according to the follow-up time while the proportion of those with below 10 mm of TST induration decreased (Figure 5A). At 24 months follow-up, none of the subjects showed <10 mm of TST induration while >15 mm of TST induration was found in 80% of spouses (Figure 5A). However, there was no significant difference in median responses to ESAT-6 in any of the cytokines tested between the groups of <10 mm and >15 mm, and none of the cytokine responses to PPD differed by the TST induration size of the spouse (data not shown). To determine the association between TST induration size and the cytokine responses to *M. tb* antigens, the TST induration sizes and cytokine responses from 101 spouses at all of the follow-up time points as well as baseline were analysed by Spearman's rank correlation test. The TST induration size did not correlate with the magnitude of the cytokine responses to either ESAT-6 or PPD stimulation (Figure 5B and 5C).

## Discussion

In this study, we examined a combination of cytokine and chemokine responses based on IFN-γ responses to ESAT-6 in spouses and TB patients in order to find potential markers which may differentiate the high risk group for *M. tb* infection from active TB patients. Our results showed that IL-10 production in response to ESAT-6 was significantly higher in ESAT-6 responder spouses (S2) than TB patients (P<0.05), and IL-17 was also highly detected in spouses (S2) compared with TB patients. In response to PPD, the ratio of IFN-γ/IL-10 and IFN-γ/IL17 were significantly lower in spouses (S2) than TB patients (P<0.01), suggesting that IL-10 and IL-17 may help to differentiate between LTBI and TB patients in addition to use of IGRA.

Many studies have been undertaken to identify alternative markers for LTBI, as current diagnostic tests such as IGRA to measure IFN-γ cannot distinguish between LTBI and active disease [13]. CXCL10 is one of the candidate markers which have been suggested to distinguish the individuals with LTBI or TB patients from uninfected controls [14] and ESAT-6 induced CXCL10 production was superior to IFN-γ in both specificity and sensitivity for detecting LTBI in rheumatoid arthritis patients who received anti-TNF-α treatment [15]. The observation of significantly higher CXCL10 production in response to ESAT-6 in spouses (S2) and TB patients compared with spouses showing negative IFN-γ responses to ESAT-6 (S1) in this study supports the previous findings that CXCL10 may be an adjunct marker for detecting infection alongside with IFN-γ although in this cohort, it could not differentiate TB contacts from TB patients.

In mice, IL-10 may play a role in disease progression. Bacterial burden was reduced in *M. tb*-infected CBA mice with decreased IL-10 expression while the mice expressing high levels of IL-10 failed to control bacteria [16]. However, in humans, lower levels of IL-10 and IL-13 have been detected in TB cases compared with contacts following PPD stimulation overnight [17]. Our study showed that levels of IL-10 and IL-13 production to PPD were not different between the spouses showing positive IFN-γ responses to ESAT-6 (>62.5 pg/mL, indicated as S2 in Figures 3 and 4) and TB patients. In contrast, ESAT-6 induced IL-10 production was significantly higher in the S2 spouses compared with TB patients (Figure 3). In addition, the ratio of IFN-γ and IL-10 in response to PPD enhanced the significance of difference between TB contacts and patients (P<0.01; Figure 4B). IL-17 enhances the Th1 memory response leading to a reduced mycobacterial burden after BCG vaccination [18] and the lower frequency of IL-17 producing cells appears to be related to TB progress [19]-[20]. Recent studies also demonstrated the potential of IL-17 to distinguish LTBI from active disease by showing significantly higher frequency of IL-17A in individuals with LTBI compared with those with active TB [21]. The results of our study are consistent with these findings; the median response of IL-17 in response to ESAT-6 was relatively lower in TB patients compared with ESAT-6 responsive TB contacts (S2) and the difference in responses between TB patients and the S2 spouses was emphasised by the ratio of IFN-γ/IL-17 in response to PPD with significantly higher median response in TB patients than the S2 spouses (P<0.01; Figure 4B).

Harari and colleagues also reported the diagnostic value of TNF-α for discriminating between latent TB and active disease by showing a substantial proportion of single positive TNF-α CD4+ T cells in response to ESAT-6 and CFP-10 in TB patients compared with the subjects with LTBI [22]. The median level of TNF-α from Quantiferon supernatants was significantly higher (P = 0.01) in TB cases than household contacts [4]. In addition, significantly higher proportions of CD4 T cells producing TNF-α alone or TNF-α, IFN-γ and IL-2 were found in TB cases compared with contacts [17], [23]. In the latter study, TNF-α production in response to PPD was also significantly higher in TB patients compared with spouses. Although it was not significantly different in response to ESAT-6 between TB patients and contacts, TNF-α production in response to ESAT-6 was higher in the S2 spouses compared with TB patients, suggesting that the level of TNF-α in response to ESAT-6 and PPD might be an additional marker for LTBI to help differentiate them from TB patients as well as uninfected controls. The IFN-γ, CXCL10 and TNF-α in response to ESAT-6 and PPD did not differentiate between LTBI (S2) and active TB in our study while significantly differential levels of the cytokines in addition to sIL2ra and MDC were detected in sera between active TB patients and individuals with TST^+^ [24], which indicates that multiplex analyses using many more cytokines in serum or culture supernatant would be of great benefit to more comprehensive biosignature for differential diagnosis of LTBI versus active TB.

Prior work in Malawi indicates that spouses of smear-positive pulmonary TB patients have intense contact with infectious TB, leading to an extremely high risk of infection [6]–[7]. The spouses in our study cohort showed a range of TST induration sizes from 0 to 68 mm with the majority of these (26/34) already showing TST induration of more than 10 mm at baseline. The percentage of spouses showing TST sizes between 10 and 15 mm was around 38% and declined throughout the follow-up period while the percentage of those with higher TST size (>15 mm) increased up to 80% at 24 months follow-up. Only 2 of 101 spouses showed TST conversion from below 10 mm to above 10 mm with an increase of more than 6 mm at 6 and 12 months follow-up, indicative of a conversion. Cytokine responses were compared between the group of below 10 mm (defined as having a lower risk of infection) and the group of above 15 mm (defined as having a higher risk of infection) but there were no significant differences in cytokine responses depending on the magnitude of the TST response (data not shown).

Previous work showing a J-shaped relationship between TB incidence rates and delayed type hypersensitivity responses to tuberculin indicates that individuals with zero TST induration may have higher risk of *M. tb* infection compared with those with an induration between 5 and 10 mm [25]. Black *et al*., reported that 13 of 270 participants with 0 mm of TST induration showed more than 500 pg/ml of IFN-γ responses to PPD, and 7 of 53 individuals with above 10 mm induration showed negative IFN-γ responses (<62.5 pg/ml) in Malawian healthy adolescents without BCG vaccination [8]. A similar pattern of responses was observed in the spouses in the current study; 3 of 5 spouses who had 0 mm of induration showed a high level of IFN-γ production to PPD (>400 pg/mL) and 16 of 91 spouses with more than 10 mm of TST induration did not produce IFN-γ or showed negative responses.

To identify a biomarker that would predict who would develop the disease among the latently infected group, ideally we could study the immune responses in the intensely exposed close TB contacts (most of whom can be assumed to have latent TB; [6]) and compare responses in those who did not develop TB with responses in those who showed disease progression during a period of follow-up. In this study, only samples from HIV negative spouses were selected for analysis, to avoid the confounding effect of HIV on the immune response. However, no HIV-negative spouses had progressed to diseases during the follow up period so no samples to test for biomarkers predictive of disease progression were available. Therefore we compared cytokine responses between TB contacts (segregated based on their IFN-γ response to ESAT-6, since the magnitude of the response may indicate bacterial load and/or risk of disease [26]) and TB patients (who, by definition have been unable to control the progress of their infection). Based on the magnitude of IFN-γ response to ESAT-6, the spouses who have been exposed to TB patients were divided into two groups; one is considered as a group of exposure with low possibility of infection (S1; <62.5 pg/mL) and the other is a group of exposure with high possibility of infection without active diseases (S2; >62.5 pg/mL). Therefore, IL-17 and IL-10 detected in S1 group are considered to be a measure of the exposure without infection while the cytokines measured in S2 group indicates a measure of the infection without disease progression.

An IGRA such as QuantiFERON TB GOLD In Tube test has been developed to diagnose *M. tb* infection by measuring the quantitative IFN-γ response to *M. tb* antigens. As the IGRA includes both ESAT-6 and CFP-10 antigens, we tested IFN-γ responses of TB patients with ESAT-6/CFP-10 stimulation. While it is possible that using ESAT-6/CFP-10 as the antigen in the culture assays for TB patients compared to ESAT-6 alone might have skewed the results, it is unlikely to have a great effect on the outcome of our analyses based on prior studies which have repeatedly shown that while ESAT-6 typically stimulates a higher percentage of responders than CFP10, the nature of the responses generated is extremely similar. When comparing responses seen to ESAT-6/CFP10 compared to responses to ESAT-6 alone, we have rarely seen more than a 3% difference in the magnitude of the response and have not observed significant differences in the type of response evoked. This observation has been confirmed by other groups showing the detailed comparison where the responses are strikingly similar [27]-[29].

In summary, almost all of the intensely-exposed contacts of smear-positive pulmonary TB patients in this study showed TST positivity but TST induration size did not correlate with cytokine responses to ESAT-6 and PPD. CXCL10, IL-17 and TNF-α production were significantly higher in the group of spouses thought to have a higher risk of *M. tb* infection based on higher ESAT-6 responses. The only single cytokines that distinguished this group of spouses from TB patients were IL-10 and IL-17. On the other hand, the ratio of IFN-γ/IL-10 and IFN-γ/IL-17 in response to PPD differentiated between TB patients and the S2 group of spouses. In conclusion, IL-17 and IL-10 may improve diagnosis of LTBI along with the IGRA. Additionally, CXCL10 and TNF-α may also have potential as markers for *M. tb* infection, and these findings may contribute to development of more accurate diagnostics for LTBI.

## Supporting Information

Figure S1 **The association of the cytokine responses between transferred and single-use samples (n = 56).** Five cytokine ELISAs were carried out with 100 µL of samples and the samples were transferred from the first to the final ELISA in the order of IL-13, IL-10 (A), CXCL10 (B), IL-17 (C) and TNF-α (D) with different volume of samples for each cytokine ELISA. All of the cytokine responses were strongly correlated between transferred and single-use samples by Spearman's rank correlation test (r>0.9, P<0.001).
(TIF)
