# Supplementary figures and images for: Combination of Cytokine Responses Indicative of Latent TB and Active TB in Malawian Adults
Source: PLoS One. 2013 Nov 18;8(11):e79742. doi: 10.1371/journal.pone.0079742 (PMC3832606; doi:10.1371/journal.pone.0079742)

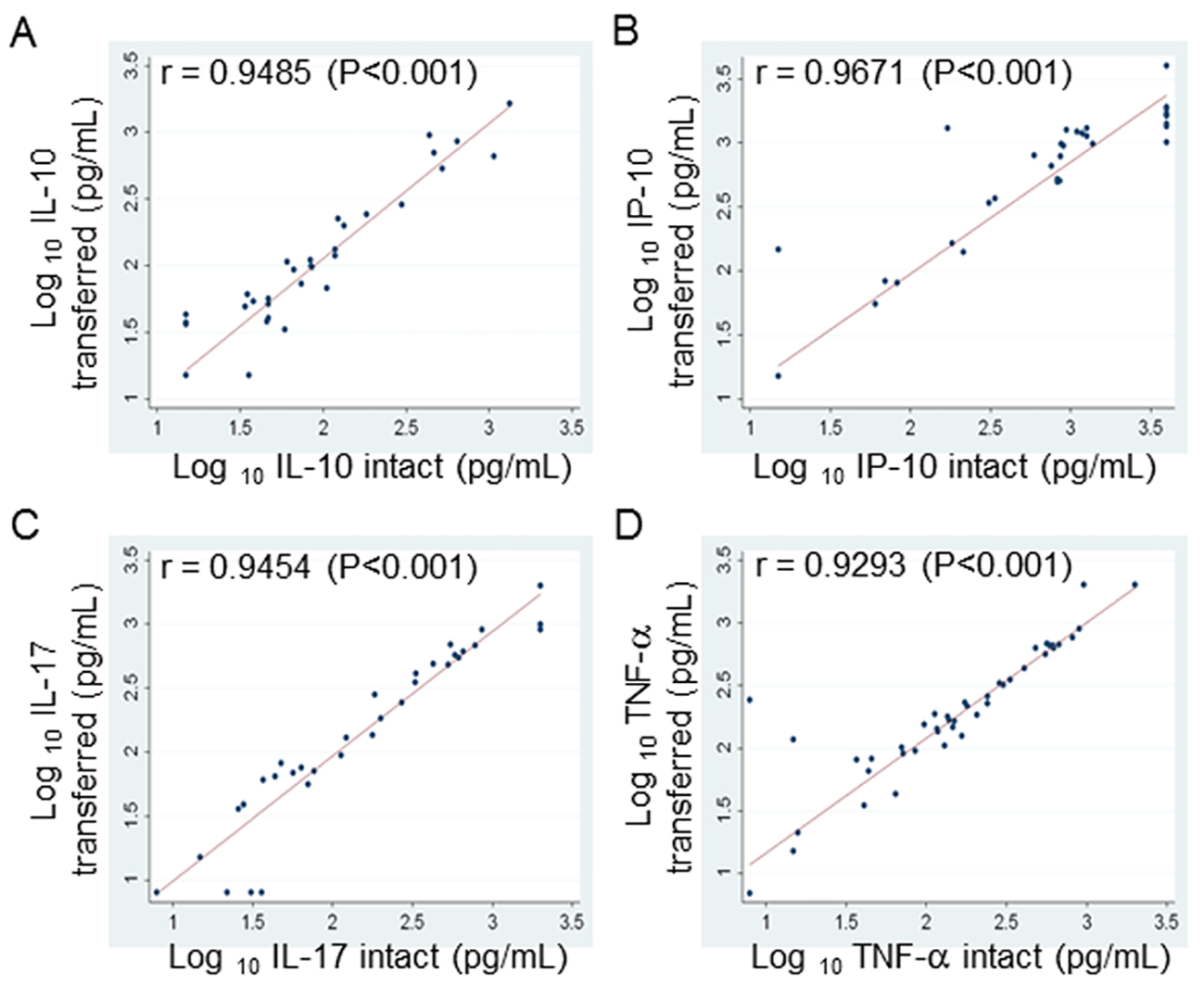

Supplement: Figure S1 — The association of the cytokine responses between transferred and single-use samples (n = 56). Five cytokine ELISAs were carried out with 100 µL of samples and the samples were transferred from the first to the final ELISA in the order of IL-13, IL-10 (A), CXCL10 (B), IL-17 (C) and TNF-α (D) with different volume of samples for each cytokine ELISA. All of the cytokine responses were strongly correlated between transferred and single-use samples by Spearman's rank correlation test (r>0.9, P<0.001). (TIF) [file pone.0079742.s001.tif]
